# The use of a neuromuscular blocking agent could significantly decrease mortality in moderate-to-severe ARDS patients: is moderate ARDS the best indication for neuromuscular blocking agents

**DOI:** 10.1186/s13054-020-02947-x

**Published:** 2020-05-13

**Authors:** Patrick M. Honore, Aude Mugisha, Luc Kugener, Sebastien Redant, Rachid Attou, Andrea Gallerani, David De Bels

**Affiliations:** ICU Department, Centre Hospitalier Universitaire Brugmann-Brugmann University Hospital, Place Van Gehuchtenplein, 4, 1020 Brussels, Belgium

We read with interest the recent meta-analysis by Chang et al. who investigated the role of neuromuscular blocking agent (NMBA) use in moderate-to-severe acute respiratory distress syndrome (ARDS) and discuss the potential mechanisms involved in the identified improvements due to the use of NMBA in ARDS [1]. They conclude that the use of NMBAs could significantly decrease mortality in moderate-to-severe ARDS patients and decrease the incidence of barotrauma during mechanical ventilation [1]. We would like to make some comments. Firstly, expert opinion challenges the conclusions of Chang et al., suggesting that NMBA use should be limited to the most hypoxemic patients (PaO2/FiO2 ratio < 120 mmHg, based on the subgroup analysis of the ACURASYS study) and not to moderate ARDS [2, 3]. The same authors also recommend that, at the early phase of mild or moderate ARDS, spontaneous breathing should be preserved [3]. Chang et al. note that NMBAs prevent patient-initiated generation of high volumes and active exhalation, facilitate patient-ventilator synchrony, provide protection from ventilator-induced lung injury (VILI), and ultimately reduce mortality in patients with moderate-to-severe ARDS [1]. The beneficial effects of NMBAs likely include not only abolition of patient-ventilator asynchronies, better lung recruitment, and decrease of VILI, but also less oxygen consumption and possible anti-inflammatory effects [3]. In addition, NMBAs are also reported to directly alleviate pulmonary and systemic inflammatory progression [4]. Other authors demonstrated that cisatracurium infusion in ARDS patients increased the end-expiratory transpulmonary pressure, contributing to the reduction of atelectrauma and expiratory derecruitment [2, 5]. We see that the conclusions of Chang et al. that the use of NMBAs could significantly decrease mortality in moderate-to-severe ARDS patients and decrease the incidence of barotrauma during mechanical ventilation are not the recommendations of the experts focusing upon the most hypoxemic patients. This message seems crucial to us, considering the numerous side effects of NMBAs.

## Data Availability

Not applicable.

## Abbreviations

NMBA: Neuromuscular blocking agent
ARDS: Acute respiratory distress syndrome
VILI: Ventilator-induced lung injury
